# Liver–kidney crosstalk in primary biliary cholangitis: insights into the mechanisms of renal involvement

**DOI:** 10.3389/fimmu.2026.1845113

**Published:** 2026-07-30

**Authors:** Zhaoyang Fan, Siqi Jia, Yi Wei, Xiaohong Fan, Xiangling Li, Li Wang

**Affiliations:** 1 Department of Nephrology, Affiliated Hospital of Shandong Second Medical University, School of Clinical Medicine, Shandong Second Medical University, Weifang, China; 2 Department of Rheumatology and Clinical Immunology, Peking Union Medical College Hospital, Chinese Academy of Medical Sciences & Peking Union Medical College, Beijing, China; 3 Department of Nephrology, Peking Union Medical College Hospital, Chinese Academy of Medical Sciences & Peking Union Medical College, Beijing, China

**Keywords:** glomerular diseases, immune-mediated renal injury, primary biliary cholangitis, renal involvement, T cell–mediated immunity, tubulointerstitial disorders

## Abstract

Primary biliary cholangitis (PBC) is a chronic autoimmune cholestatic liver disease primarily affecting the intrahepatic small bile ducts. Although PBC is mainly a hepatobiliary disease, renal abnormalities have been reported in a subset of patients and should be regarded as uncommon but clinically relevant extrahepatic manifestations. Reported glomerular lesions in patients with PBC include membranous nephropathy, immunoglobulin A nephropathy, crescentic glomerulonephritis, and minimal change disease, although most evidence is derived from case reports and small series. Tubulointerstitial involvement has also been described, most commonly as distal renal tubular acidosis in reported cases and less commonly as Fanconi syndrome; these conditions may reflect immune-mediated tubular dysfunction in selected patients. In addition, metabolic disturbances secondary to cholestasis and tubular injury may increase the risk of nephrolithiasis and nephrocalcinosis, whereas advanced cirrhosis can precipitate hepatorenal syndrome. Limited reports further suggest that cholestatic nephropathy and drug-related nephrotoxicity may contribute to renal impairment in selected patients. From a mechanistic perspective, immune complex deposition, T-cell–mediated inflammation, mitochondrial dysfunction, cholestatic/metabolic disturbances, and gut microbiota dysbiosis have been proposed as potential contributors, but direct causal evidence remains limited. Given the heterogeneity and potential clinical significance of renal manifestations in PBC, early detection, thorough nephrological assessment, and individualized therapeutic strategies are essential.

## 1 Introduction

Primary biliary cholangitis (PBC) is a chronic autoimmune cholestatic liver disease characterized by non-suppurative, progressive destruction of the intrahepatic small bile ducts. The fundamental pathological process involves immune-mediated injury to biliary epithelial cells, leading to persistent cholestasis, progressive fibrosis, and ultimately cirrhosis (1). PBC predominantly affects middle-aged and elderly women. Ursodeoxycholic acid (UDCA) remains the first-line therapy; however, approximately one-third of patients exhibit an inadequate biochemical response, which is associated with continued disease progression and a markedly increased risk of hepatic decompensation and liver-related mortality (2, 3). The epidemiological burden of PBC has increased in recent decades, with recent studies reporting rising prevalence worldwide despite geographical heterogeneity (4). Although the precise pathogenesis of PBC remains incompletely understood, current evidence supports a multifactorial etiology involving genetic susceptibility, immune dysregulation, environmental triggers, and alterations in the intestinal microenvironment (5, 6). Clinically, patients commonly present with pruritus and fatigue, accompanied by biochemical abnormalities related to cholestasis. With advancing disease, features of portal hypertension and cirrhosis may develop (6–8).

Traditionally, PBC has been regarded as an organ-specific autoimmune disease confined to the intrahepatic bile ducts. However, accumulating evidence suggests that PBC represents a systemic autoimmune disorder with extrahepatic involvement affecting multiple organ systems, including the respiratory, gastrointestinal, urinary, and hematologic systems. Reported extrahepatic manifestations include pulmonary arterial hypertension, interstitial lung disease, hemolytic anemia, and renal impairment (1, 9–11). Renal involvement is well recognized in systemic autoimmune diseases, including systemic lupus erythematosus (SLE), Sjögren disease (SjD; formerly known as Sjögren syndrome), systemic sclerosis, and antineutrophil cytoplasmic antibody (ANCA)–associated vasculitis (AAV); however, it remains less well characterized and underrecognized in PBC. PBC also frequently coexists with other autoimmune diseases, among which SjD is one of the most common comorbidities. A systematic review and meta-analysis reported that the prevalence of SjD in patients with PBC varied widely across studies, with a pooled estimate of approximately 35% (12). This comorbidity is clinically relevant because renal involvement is a recognized manifestation of SjD. A recent multicenter retrospective study of 1, 058 patients with PBC reported a baseline prevalence of chronic kidney disease of 10% and a cumulative incidence of 7% during follow-up (13). Despite this emerging cohort-based evidence, the current literature on PBC-associated renal involvement remains limited, consisting largely of case reports and small retrospective studies, and standardized diagnostic and therapeutic approaches have not yet been established.

**Figure 1:**
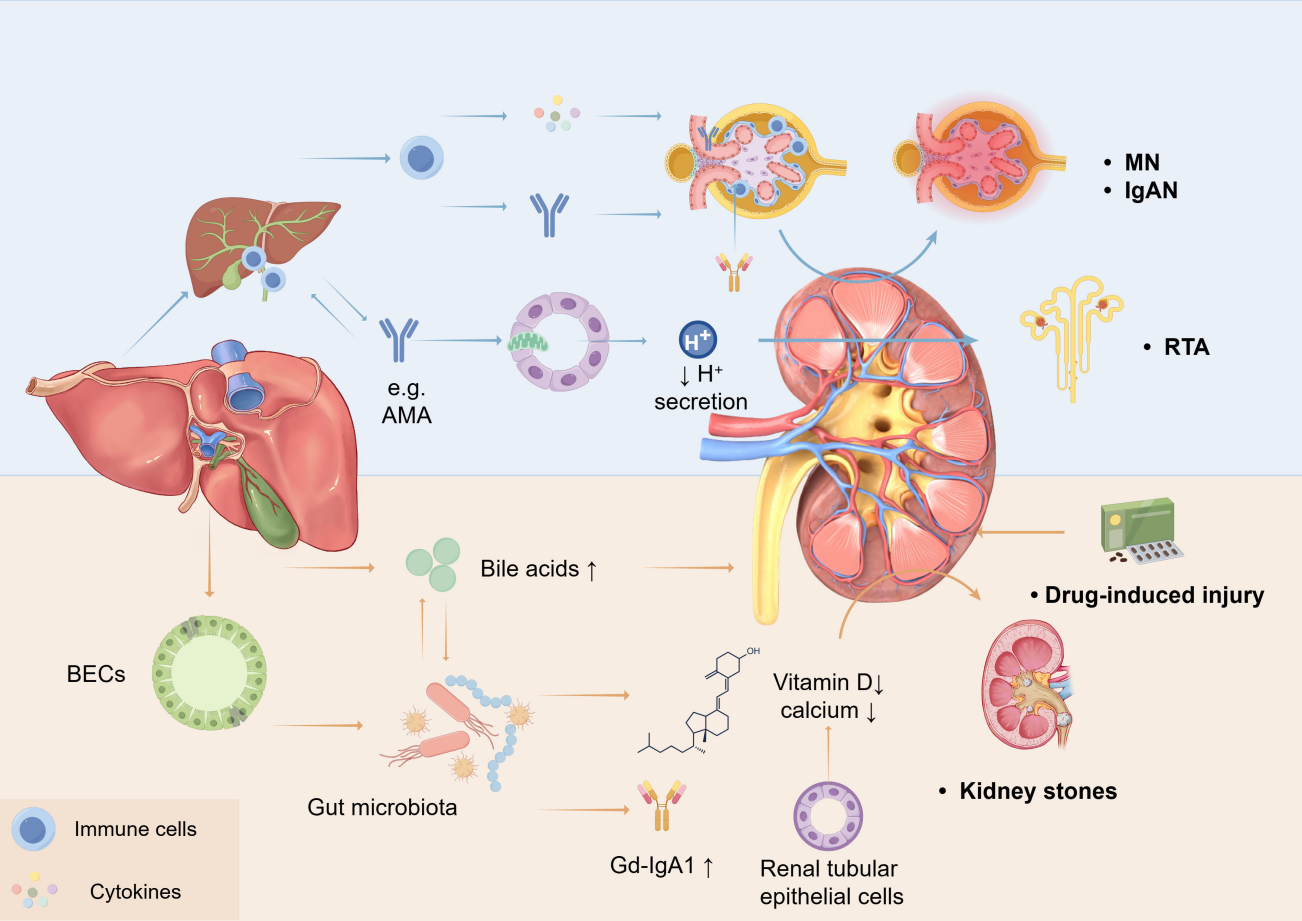
Spectrum and pathophysiological mechanisms of renal involvement in primary biliary cholangitis. Conceptual model of reported renal manifestations in primary biliary cholangitis (PBC). Immune dysregulation and cholestatic/metabolic disturbances may contribute to renal abnormalities in selected patients. Autoantibodies (e.g., AMA), cytokines, and immune complexes contribute to glomerular injury, leading to membranous nephropathy (MN) and IgA nephropathy (IgAN). Immune-mediated tubular damage may impair H^+^ secretion and result in renal tubular acidosis (RTA). In parallel, elevated bile acids, gut microbiota dysbiosis, and increased galactose-deficient IgA1 may be associated with immune activation and mesangial deposition, although direct causal links in PBC remain to be established. Metabolic alterations, including reduced vitamin D and calcium levels, along with bile acid–mediated toxicity and drug-induced injury, further exacerbate renal damage.

Accordingly, this review aims to comprehensively summarize the clinical features, potential pathophysiological mechanisms, and management strategies of renal involvement in PBC. Through this review, we aim to enhance clinicians’ awareness of renal involvement in patients with PBC and to provide a reference framework for future research in this field (**Figure 1**).

## 2 Reported renal manifestations in PBC

### 2.1 Glomerular diseases

The kidneys receive approximately 20–25% of cardiac output, reflecting their high perfusion state. The glomerulus consists of a dense capillary network exposed to high blood flow and filtration pressure, making it particularly susceptible to the deposition of circulating immune complexes. Its physiological features—namely high perfusion, high filtration capacity, propensity for immune complex trapping, and amplification of inflammatory cascades—facilitate the retention of circulating immune complexes and autoantibodies, thereby initiating localized inflammatory responses (14). Moreover, the glomerular filtration barrier is a highly specialized and delicate trilaminar structure consisting of fenestrated endothelial cells, the glomerular basement membrane (GBM), and podocytes. Disruption of either its charge-selective properties or structural integrity enhances the deposition and persistence of immune mediators. The GBM and mesangial matrix are enriched with diverse autoantigenic components, providing additional targets for autoantibody binding and immune complex formation (15). Therefore, in the setting of systemic autoimmunity, the glomerulus is often one of the most commonly and prominently affected renal structures.

Lupus nephritis (LN) represents a prototypical immune complex–mediated glomerular disease, in which immune complex deposition activates complement and promotes glomerular inflammation (16). In contrast, ANCA-associated vasculitis is characterized by pauci-immune necrotizing and crescentic glomerulonephritis driven by ANCA-activated neutrophils (17).

Taken together, as a key target organ in systemic immune dysregulation, the glomerulus is highly vulnerable to injury across a spectrum of autoimmune diseases. Based on the available case reports and small series, the most frequently reported glomerular lesions in patients with PBC appear to be membranous nephropathy (MN), followed by IgA nephropathy (IgAN) and other less common forms of glomerular injury. The following sections summarize these patterns and discuss their possible clinical and immunopathological significance.

#### 2.1.1 Membranous nephropathy

##### 2.1.1.1 Clinical evidence

Among glomerular lesions reported in patients with PBC, MN appears to be the most frequently described pattern in the available literature. However, current evidence remains limited to case reports and small retrospective series. A review of the available literature identified 12 reported cases of PBC complicated by MN, with clinical characteristics summarized in **Table 1**. Among these patients, five initially presented with edema accompanied by abnormal liver biochemistry. Three were first diagnosed with PBC based on abnormal liver enzyme levels and positive autoantibodies and subsequently developed bilateral lower extremity edema and MN-related manifestations during follow-up. The original reports did not clearly describe the sequence of onset between PBC and renal disease in the remaining four patients (18–24).

**Table 1:** Clinical features and treatment strategies in primary biliary cirrhosis patients with membranous nephropathy.

Source article	Age (year) /sex	PLA2R	Cr (μmol/L)	24hUP (g)	Treatment of glomerulone-phritis	At last follow-up	
						Cr (μmol/L)	24hUP (g)
Bian, Sainan et al. (18)	50/F	–	54	3.6	Prednisone	53	0.7
Bian, Sainan et al. (18)	65/F	–	114	13.4	Prednisone, CTX	58	0.56
Bian, Sainan et al. (18)	49/F	+	48	6.9	ARB	49	0.2
Bian, Sainan et al. (18)	52/F	+	176	8.3	Prednisone, CTX, Rituximab	264	7.0
Lei, Xiwen et al. (19)	53/M	+	/	8.2	Methylprednisolone, Rituximab	/	3.2
Sakamaki, Yusuke et al. (20)	76/M	/	88.4	10.1	CsA	88.4	0.68
Zimmermann, Jonas et al. (21)	39/M	–	79.6	7.3	Prednisone, CsA	114.9	2.0
Sato, Shuzo et al. (22)	40/M	/	57.5	4.45	ARB	/	4.0
Hong, Xia et al. (23)	54/M	+	64.5	5.11	Prednisone, AZA	102	2.6
Dauvergne, Maxime et al. (24)	55/F	–	58.7	1.6	Prednisone, MMF	/	/
Dauvergne, Maxime et al. (24)	66/M	–	480	11	Prednisone, AZA	/	/
Dauvergne, Maxime et al. (24)	64/M	+	70	3	Prednisone	/	/

F, female; M, male; PLA2R, phospholipase A2 receptor; Cr, creatinine; 24hUP, 24-h urine protein; CTX, cyclophosphamide; ARB, angiotensin receptor blocker; CsA, cyclosporine A, AZA, azathioprine; MMF, mycophenolate mofetil.

##### 2.1.1.2 Pathological/diagnostic features

MN is an immune-mediated glomerular disease characterized by subepithelial immune complex deposition along the GBM. Its pathogenesis primarily involves complement activation and podocyte injury, resulting in disruption of the glomerular filtration barrier and diffuse thickening of the GBM. Histopathologically, MN is characterized by thickened glomerular capillary walls with “spike” formation on silver staining, granular deposition of IgG and complement components along the capillary loops on immunofluorescence, and subepithelial electron-dense deposits observed on electron microscopy (25). In 2009, Beck et al. identified podocyte-expressed PLA2R as a major target antigen in adult MN. Circulating autoantibodies against PLA2R were subsequently detected in the serum of affected patients, establishing PLA2R as a central antigen in the pathogenesis of primary MN (26). These observations raise an important question: does MN in the context of PBC represent idiopathic MN that coincidentally occurs in patients with PBC, or does it constitute a secondary manifestation driven by the underlying immune dysregulation of PBC? To address this issue, particular attention should be directed toward the expression of anti-phospholipase A2 receptor (PLA2R) antibodies and the immunopathological features of affected patients.

##### 2.1.1.3 Possible mechanisms

###### 2.1.1.3.1 PLA2R antibody–positive MN

The immune response in PLA2R-associated MN is characterized predominantly by IgG4 autoantibodies targeting PLA2R on the podocyte surface. Binding of these antibodies results in *in situ* immune complex formation beneath the podocyte layer and activation of the complement cascade—particularly the assembly of the C5b-9 membrane attack complex. This leads to podocyte injury, cytoskeletal disruption, and increased glomerular permeability, ultimately manifesting as proteinuria and nephrotic syndrome (27). In recent years, several additional target antigens have been identified in MN, including neural epidermal growth factor-like 1 protein (NELL1), protocadherin 7 (PCDH7), and exostosin 1/2 (EXT1/EXT2). These antigens are predominantly localized to podocytes or the GBM and similarly result in subepithelial immune complex deposition. Currently, there is no direct evidence demonstrating that these antigens specifically target hepatocytes or biliary epithelial cells. Notably, EXT1/EXT2-associated MN is frequently observed in patients with systemic autoimmune diseases such as SjD and SLE, suggesting a potential link to systemic immune dysregulation (28, 29). Although future studies may clarify possible associations between these novel antigens and autoimmune liver diseases, including PBC, definitive mechanistic and clinical evidence is currently lacking. Therefore, in patients with PBC who are positive for anti-PLA2R antibodies, MN is more likely to represent a coincidental overlap with primary (idiopathic) MN rather than a direct secondary manifestation of PBC-related immune abnormalities.

###### 2.1.1.3.2 PLA2R antibody–negative MN

In contrast, PLA2R-negative MN occurring in patients with PBC may be more plausibly associated with systemic immune dysregulation, although current evidence is insufficient to establish a direct causal relationship with PBC. The characteristic hepatic pathology of PBC includes portal tract infiltration by activated T lymphocytes and upregulation of T-cell surface markers, indicating that T-cell–mediated immune responses and cytokine release are central drivers of disease progression.In reported cases of MN overlapping with anti-GBM disease, renal biopsy demonstrated multifocal lymphocytic and mononuclear cell infiltration, reflecting active intrarenal inflammation. Such inflammatory infiltrates may amplify glomerular injury by establishing a pro-inflammatory microenvironment and releasing multiple cytokines. T-cell–mediated cellular immunity may therefore contribute to the initiation or propagation of glomerular damage in this context. Additionally, PBC is frequently associated with elevated serum IgM levels. It has been hypothesized that circulating immune components—such as IgM or anti-mitochondrial antibody subtype M2 (AMA-M2)—may deposit along the GBM, contributing to or exacerbating MN-like lesions. Previous case reports have described granular IgM deposition along glomerular capillary walls accompanied by subepithelial electron-dense deposits on electron microscopy (30, 31). Among the 11 patients who underwent renal biopsy in the reviewed cases, two exhibited IgM deposition in addition to IgG and C3. These findings raise the possibility that IgM-mediated immune deposition within the GBM may contribute to MN-like lesions in selected patients with PBC, although this mechanism remains speculative.

##### 2.1.1.4 Management

Management of MN generally depends on the severity of proteinuria, renal function, anti-PLA2R antibody status, and risk of progression. Standard therapeutic strategies for MN include glucocorticoids combined with cyclophosphamide (CTX), calcineurin inhibitors such as cyclosporine (CsA), and B-cell–depleting agents including rituximab. In the reviewed cases, the majority of patients (9/12, 75%) received corticosteroid therapy, with several undergoing combination immunosuppressive treatment involving CTX, CsA, or rituximab for induction of remission. Based on available follow-up data, all patients demonstrated improvement in proteinuria, achieving either complete or partial remission of renal involvement.

For patients diagnosed with both PBC and MN, optimal clinical management requires careful differentiation between primary and secondary MN. Renal biopsy combined with serological testing—particularly assessment of anti-PLA2R antibodies—is essential for clarifying the underlying pathogenic mechanism. Accurate classification enables individualized therapeutic decision-making and may improve renal and hepatic outcomes.

Overall, MN is the most frequently reported glomerular lesion in PBC, but available evidence is insufficient to determine whether this association is causal or coincidental in most patients.

#### 2.1.2 IgA nephropathy

##### 2.1.2.1 Clinical evidence

Reports of PBC complicated by IgAN remain exceedingly scarce and are limited to isolated case descriptions. The following discussion is based on two published cases (32, 33). Both patients were middle-aged or elderly women with concurrent PBC and IgAN, and both had additional autoimmune comorbidities, such as rheumatoid arthritis. Renal biopsy in each case confirmed mesangial IgA deposition. Notably, one patient also exhibited IgM-positive plasma cell–associated tubulointerstitial nephritis (IgM^+^PC-TIN), suggesting that renal involvement in PBC may extend beyond the glomerulus to include tubulointerstitial compartments, thereby producing heterogeneous and complex clinical phenotypes.

##### 2.1.2.2 Pathological/diagnostic features

The central pathogenic mechanism of IgAN involves the mesangial deposition of immune complexes containing galactose-deficient IgA1 (Gd-IgA1), which subsequently trigger localized inflammatory and proliferative responses within the glomerulus (34). Histopathologically, light microscopy typically demonstrates diffuse mesangial hypercellularity and mesangial matrix expansion, which may present in diffuse or focal segmental patterns. Immunofluorescence microscopy constitutes the diagnostic hallmark, revealing granular or clumped mesangial deposits of IgA—often IgA-dominant—frequently accompanied by complement component C3 deposition (35). These pathological features remain essential for confirming IgAN in patients with PBC who present with hematuria, proteinuria, or impaired renal function.

##### 2.1.2.3 Possible mechanisms

Despite involving distinct primary target organs, PBC and IgAN appear to share overlapping immunopathological mechanisms, particularly T-cell–mediated adaptive immune dysregulation. In PBC, autoreactive T lymphocytes selectively target biliary epithelial cells, while impaired regulatory T-cell function may further enhance anti-mitochondrial antibody production and perpetuate bile duct destruction. In IgAN, substantial CD3^+^ T-cell infiltration is frequently observed in renal tissue, and effector T-cell subsets, such as T helper 1 (Th1) and T helper 17 (Th17) cells, are believed to exacerbate glomerular injury through the release of proinflammatory cytokines (36). These observations suggest that dysregulated cellular immunity may represent a shared pathogenic axis.

Increasing evidence also implicates gut microbiota dysbiosis in the pathogenesis of PBC. Epidemiological studies have identified prior Escherichia coli infection and recurrent urinary tract infections as potential environmental triggers. Patients with PBC exhibit reduced abundance of beneficial commensal bacteria, including Bifidobacterium and Bacteroides species, along with decreased overall microbial diversity compared to healthy controls (37). Similarly, gut dysbiosis has been implicated in the pathogenesis of IgAN. Disruption of intestinal barrier integrity enhances antigen exposure and stimulates mucosal immune activation. Through Toll-like receptor (TLR) signaling and cytokine-mediated pathways—particularly involving B-cell activating factor (BAFF) and a proliferation-inducing ligand (APRIL)—B cells undergo class-switch recombination and produce excessive Gd-IgA1. These aberrantly glycosylated IgA1 molecules form circulating immune complexes that deposit in the glomerular mesangium, initiating renal injury (38). Taken together, disruption of the intestinal microenvironment may provide a conceptual link between PBC and IgAN within the proposed “gut–liver–kidney axis,” although direct evidence in patients with PBC–IgAN overlap remains limited. Within this framework, gut dysbiosis may contribute to hepatic immune activation in PBC and mucosal IgA dysregulation in IgAN; however, whether it directly drives renal injury in PBC remains unproven. The systemic immune dysregulation characteristic of PBC may further predispose to immune complex deposition and renal inflammation.

Therapeutic strategies targeting the gut–immune interface have garnered increasing attention. Budesonide, an orally administered targeted-release corticosteroid formulation, enables site-specific delivery within the intestinal mucosa. By suppressing aberrant mucosal immune activation and inflammatory mediator production, it may reduce Gd-IgA1 generation and downstream immune complex formation. Owing to its predominantly local intestinal activity and reduced systemic bioavailability, budesonide offers a potentially favorable safety profile. This therapeutic approach may represent a promising direction for patients with PBC complicated by IgAN, although clinical validation remains necessary.

In summary, although current evidence is limited, the coexistence of PBC and IgAN may be interpreted within a hypothesis-generating immune-metabolic framework centered on the gut–liver–kidney axis. Gut microbiota dysbiosis may represent a shared background factor associated with hepatic autoimmunity and aberrant IgA production, rather than a proven initiating cause. Subsequent systemic immune dysregulation may facilitate immune complex deposition and renal inflammation. While this hypothesis provides a plausible immunological explanation for their coexistence, robust mechanistic investigations and prospective clinical studies are required to substantiate this proposed pathogenic linkage.

##### 2.1.2.4 Management

Because only isolated cases have been reported, no PBC-specific treatment strategy for IgAN has been established. Management should be guided by the severity of renal involvement, histopathological findings, and the presence of concomitant autoimmune diseases. In the reported cases, glucocorticoid therapy was associated with improvement in renal function and proteinuria. Therapeutic strategies targeting the gut–immune interface, including targeted-release corticosteroids, are biologically plausible in IgAN, but their role in patients with PBC complicated by IgAN remains speculative and requires clinical validation.

Therefore, the coexistence of PBC and IgAN should currently be interpreted as a rare reported overlap, with the gut–liver–kidney axis serving as a conceptual framework rather than a proven causal pathway.

#### 2.1.3 Other glomerular diseases

Previous reports indicate that, beyond MN and IgAN, PBC may also be associated with a spectrum of additional glomerular lesions. In 1992, Bissuel et al. described a patient with PBC complicated by pulmonary hemorrhage and focal proliferative glomerulonephritis, highlighting the potential for severe immune-mediated glomerular injury in the setting of PBC (39). Subsequently, in 2007, Lai et al. reported a case of PBC associated with minimal change disease. The patient initially responded well to glucocorticoid therapy but experienced relapse during steroid tapering, suggesting an immune-dependent disease course (40). Furthermore, isolated reports have documented the development of PBC following episodes of acute glomerulonephritis, implying a potentially complex and bidirectional relationship between hepatic and renal autoimmunity within a shared systemic immune context (41).

Collectively, these observations underscore the heterogeneity of glomerular diseases reported in association with PBC. The underlying renal pathology may reflect immune complex–mediated glomerulonephritis, renal manifestations of overlapping systemic autoimmune disorders (such as ANCA-associated vasculitis), or coincidental coexistence with post-infectious nephritis. This diversity highlights the need for careful clinical and pathological differentiation.

Accordingly, in PBC patients presenting with unexplained hematuria, proteinuria, or renal function impairment, a comprehensive nephrological evaluation is warranted. Particular attention should be paid to the possibility of underlying glomerular disease, especially when urinary abnormalities are accompanied by active sediment or significant proteinuria. When clinically appropriate, renal biopsy should be strongly considered to clarify the histopathological subtype and underlying mechanism of glomerular injury, thereby facilitating accurate diagnosis and guiding individualized therapeutic strategies.

### 2.2 Tubulointerstitial diseases

The renal tubules play a critical role in regulating body fluid composition, maintaining electrolyte balance, and ensuring acid-base homeostasis. They process primary urine through key functions such as reabsorption, secretion, and excretion. Consequently, damage to the tubules often results in metabolic disturbances and electrolyte imbalances. In patients with PBC, the primary clinical manifestations of tubular injury include distal renal tubular acidosis (dRTA) and Fanconi syndrome, both of which are secondary to tubulointerstitial nephritis (TIN). Among these, Fanconi syndrome due to TIN is relatively uncommon in PBC. This review systematically examines the clinical manifestations, pathogenesis, and diagnostic and therapeutic advances related to PBC complicated by dRTA and Fanconi syndrome.

#### 2.2.1 Renal tubular acidosis

##### 2.2.1.1 Clinical evidence

RTA appears to be one of the better-described tubulointerstitial abnormalities reported in patients with PBC, although the number of published cases remains small. A review of the literature identified six reported cases of PBC complicated by RTA (42–44). All patients were middle-aged women, consistent with the typical demographic profile of PBC. In five cases, abnormalities in routine urinalysis emerged during follow-up after the diagnosis of PBC, whereas in one case, abnormal urinary findings led to the concurrent identification of PBC.

##### 2.2.1.2 Pathological/diagnostic features

RTA is a metabolic acidosis resulting from impaired renal acid excretion. It is characterized by hyperchloremic metabolic acidosis with a normal anion gap, typically accompanied by a normal or mildly impaired glomerular filtration rate. RTA commonly presents with hypokalemia, medullary calcification, and renal calculi.

The predominant clinical features included normal anion gap hyperchloremic metabolic acidosis, refractory hypokalemia, and impaired urinary acidification. A key diagnostic hallmark was the inability to appropriately acidify the urine, with urinary pH persistently exceeding 5.5 despite the presence of systemic metabolic acidosis—findings consistent with dRTA. Although PBC frequently coexists with SjD, a well-recognized cause of dRTA, all reported patients tested negative for anti-SSA and anti-SSB antibodies. These serological results do not support the diagnosis of concomitant SjD. After excluding SjD, which frequently coexists with PBC and is a well-recognized cause of dRTA, the tubular dysfunction observed in these patients is more likely attributable to PBC itself.

##### 2.2.1.3 Possible mechanisms

Pathological and immunohistochemical findings from reported cases suggest that autoimmune injury to renal tubular epithelial cells may contribute to dRTA in selected patients. Key proteins, including the basolateral AE1 anion exchanger and apical H^+^-ATPase, were completely absent. This pattern is consistent with dRTA seen in SjD, suggesting shared autoimmune targets (45). Additionally, the detection of autoantibodies in patient serum that bind to healthy renal collecting duct cells further supports the “molecular mimicry” hypothesis. This suggests that PBC-associated AMA or other unknown antibodies may cross-react with mitochondria-rich renal tubular epithelial cells, ultimately leading to tubular dysfunction (46). Thus, this renal complication represents a localized manifestation of systemic autoimmune activity within the renal tubules. However, these observations are based on limited cases, and the precise renal autoantigens remain undefined.

##### 2.2.1.4 Management

Management should first focus on correction of metabolic acidosis and electrolyte abnormalities, typically with oral potassium citrate or sodium bicarbonate. Active vitamin D and calcium supplementation may be considered when chronic acidosis, phosphate wasting, or bone disease is present. Glucocorticoids may be considered in selected patients with biopsy-supported active immune-mediated tubulointerstitial injury, but their use should be individualized because standardized treatment recommendations for PBC-associated dRTA are lacking.

Thus, dRTA may represent an immune-mediated tubular abnormality in selected patients with PBC, but careful exclusion of SjD and other causes remains essential.

#### 2.2.2 Fanconi syndrome

##### 2.2.2.1 Clinical evidence

Fanconi syndrome represents a rare but clinically important form of tubular dysfunction reported in patients with PBC. Available evidence is limited to isolated case reports (47, 48). Reported patients presented with severe bone pain, pathological fractures, or electrolyte abnormalities, and laboratory findings may include hypophosphatemia, hypouricemia, glycosuria, aminoaciduria, and metabolic acidosis.

##### 2.2.2.2 Pathological/diagnostic features

Renal biopsy may demonstrate tubulointerstitial injury with inflammatory cell infiltration, supporting the diagnosis of tubulointerstitial nephritis-associated Fanconi syndrome. Assessment should focus on confirming proximal tubular dysfunction and excluding alternative causes, including drugs, monoclonal gammopathy, inherited tubular disorders, and other autoimmune diseases.

##### 2.2.2.3 Possible mechanisms

The mechanism linking PBC to Fanconi syndrome remains uncertain. It has been hypothesized that cross-reactive immune responses against mitochondria-rich tubular epithelial cells, possibly involving AMA or related autoantibodies, may impair mitochondrial function and ATP production in proximal tubular cells.

In reported cases, renal biopsy has shown significant lymphocytic infiltration, a definitive pathological feature shared between TIN and PBC. In PBC, hepatic bile duct epithelial cells express specific autoantigens (e.g., mitochondrial antigen PDC-E2), and similar antigens may also be present on renal tubular epithelial cells. This promotes the accumulation of T lymphocytes (CD4^+^, CD8^+^, CD3^+^) in the renal interstitium, leading to tubulointerstitial injury and ultimately resulting in Fanconi syndrome. Research indicates that Fanconi syndrome and TIN are typical renal manifestations of mitochondrial diseases (46). Moreover, elevated AMA in PBC patients may circulate to the kidneys, disrupting mitochondrial function in tubular epithelial cells and inhibiting ATP production. As the reabsorption processes in the proximal tubules are highly energy-dependent, impaired ATP synthesis leads to the failure of multiple transport functions, thereby inducing Fanconi syndrome and associated TIN. However, this proposed mechanism remains speculative and requires further validation.

##### 2.2.2.4 Management

In summary, the presence of urinary casts, glycosuria, hypokalemia, or other electrolyte disturbances in PBC patients suggests potential tubular injury. Renal biopsy may be considered when the cause is unclear, renal dysfunction is progressive, or active immune-mediated tubulointerstitial nephritis is suspected. Treatment should follow a three-pronged strategy: correction of acidosis and electrolyte imbalances, prevention of osteoporosis, and immunosuppressive therapy when renal pathology indicates active immune cell infiltration. In such cases, glucocorticoids may be considered as an additional therapeutic option.

Fanconi syndrome should therefore be regarded as a rare reported tubular manifestation rather than a typical renal complication of PBC.

### 2.3 Metabolic/hemodynamic-related renal pathology

Beyond immune-mediated mechanisms, renal complications in PBC may result from a combination of coexisting disorders, disease-related metabolic derangements, hemodynamic changes, and the long-term impact of persistent cholestasis. Compared with glomerular and tubular lesions, these manifestations are less frequently reported and remain less clearly defined.

#### 2.3.1 Renal calcification and nephrolithiasis

Nephrolithiasis and nephrocalcinosis appear to be extremely rare in patients with PBC, with only isolated case reports available in the literature. Therefore, the following discussion should be interpreted as a mechanistic hypothesis based on limited clinical observations rather than as evidence that nephrolithiasis is a typical renal manifestation of PBC. Previous studies have reported that patients with autoimmune hepatitis (AIH) may exhibit hypercalciuria and an increased risk of nephrolithiasis. In conjunction with the aforementioned case of PBC-associated RTA in which bilateral nephrocalcinosis was observed, these findings raise the possibility that PBC may be associated with disturbances in calcium–phosphorus metabolism (49, 50). Nevertheless, renal calcification and kidney stones are not considered primary clinical manifestations of PBC. Rather, they are more likely secondary complications arising from renal tubular dysfunction or intestinal metabolic alterations. At the renal level, PBC-associated tubular injury—particularly dRTA—may facilitate calcium salt deposition and stone formation. Persistent urinary alkalinization reduces calcium phosphate solubility, while concomitant hypercalciuria and hypocitraturia further promote crystallization and intrarenal calcium deposition (51). At the intestinal level, chronic cholestasis may result in fat malabsorption. Unabsorbed fatty acids bind luminal calcium, thereby reducing the availability of calcium to complex with oxalate in the gut. This increases free oxalate absorption, leading to hyperoxaluria and a heightened risk of calcium oxalate stone formation (52). Therefore, in PBC patients presenting with nephrolithiasis or nephrocalcinosis, clinical management should extend beyond symptomatic stone treatment and instead prioritize comprehensive evaluation and correction of underlying metabolic derangements and tubular dysfunction. Addressing these pathophysiological mechanisms may be critical for preventing recurrence and improving long-term renal outcomes.

#### 2.3.2 Hepatorenal syndrome

Progressive destruction of bile ducts leads to periportal inflammation and fibrosis, eventually progressing to cirrhosis and its associated complications in a subset of patients. Historical studies have suggested that untreated patients with PBC have a substantially increased risk of hepatic decompensation (53). Since its introduction in the early 1990s, UDCA has become the cornerstone of PBC therapy, effectively improving biochemical markers, delaying histological progression, postponing the onset of esophageal varices, and reducing the need for liver transplantation. However, approximately 40% of PBC patients exhibit inadequate biochemical response to UDCA. This inadequate biochemical response is associated with an increased long-term risk of disease progression rather than uniformly poor prognosis (54). Importantly, contemporary data suggest that hepatic decompensation and poor survival in PBC are largely concentrated in patients with advanced chronic liver disease and clinically significant portal hypertension, whereas patients without these features generally have favorable outcomes (8). In end-stage decompensated cirrhosis, significant disruption of systemic circulation and renal hemodynamics can lead to severe renal impairment, ultimately triggering hepatorenal syndrome (HRS). Although HRS is relatively uncommon in PBC, its diagnosis is complex, treatment is challenging, and prognosis is poor. Liver transplantation remains the only curative intervention for HRS; when transplantation is not feasible, comprehensive management primarily relies on vasoconstrictors combined with intravenous albumin infusion (55).

#### 2.3.3 Bile-associated kidney injury

In PBC, impaired bile excretion leads to chronic cholestasis and systemic accumulation of bile acids and bilirubin, which may exert direct cytotoxic effects on renal tubular epithelial cells. Through membrane disruption, oxidative stress, and mitochondrial injury, these substances contribute to tubular dysfunction, a condition termed cholestatic nephropathy (CN) (56). Bile cast formation within the tubular lumen may further aggravate obstruction and inflammation. Clinically, CN presents with nonspecific features of acute or subacute kidney injury, and definitive diagnosis relies on renal biopsy demonstrating characteristic intratubular bile casts, along with elevated serum bile acids and bilirubin levels.

Although cases of renal injury secondary to severe cholestasis and hyperbilirubinemia have been documented, no definitive reports have directly attributed cholestatic nephropathy specifically to PBC-related bile toxicity (57, 58). However, bile-associated kidney injury is extremely rare in PBC and is usually described in patients with very high bilirubin levels, commonly exceeding 20 mg/dL; such levels are rarely observed in patients with PBC unless they have end-stage or severely decompensated liver disease (59, 60). Therefore, CN should not be regarded as a typical renal manifestation of PBC, but rather as a rare potential complication in the setting of advanced cholestasis or end-stage liver disease. At present, there is no disease-specific therapy for CN. Management primarily focuses on alleviating cholestasis, reducing circulating bile acid and bilirubin levels, and providing supportive renal care. Early recognition and timely intervention targeting the underlying hepatobiliary disorder are essential to prevent progressive tubular injury and deterioration of renal function.

### 2.4 Drug-induced kidney injury

Early and standardized medication is crucial for delaying the progression of PBC, but a comprehensive assessment of renal safety is essential when selecting drugs. Recent studies suggest that UDCA, a first-line treatment for PBC, may exert renal protective effects. Its mechanism involves activating peroxisome proliferator-activated receptor-gamma (PPARγ), which enhances fatty acid oxidation, promotes ATP production, and reduces lipid accumulation in proximal tubular epithelial cells, thereby mitigating renal ischemia-reperfusion injury (61). While second-line treatments such as fenofibrate and bezafibrate may improve biochemical markers, their potential renal effects warrant attention. Fenofibrate has been associated with transient, reversible elevations in serum creatinine. Although bezafibrate significantly reduced alkaline phosphatase (ALP) and improved symptoms in a Phase III clinical trial (400 mg/day, 24 months), it was also linked to an average increase in serum creatinine of approximately 5% from baseline, suggesting potential renal toxicity in some patients (62, 63). With advances in PBC treatment, novel agents such as elafibranor and seladelpar have emerged, though their long-term adverse reaction profiles are yet to be fully characterized (64, 65). Therefore, enhanced renal monitoring is critical to balance efficacy and safety throughout PBC therapy.

## 3 Summary and outlook

This review systematically examines the clinical manifestations, pathogenesis, and management strategies for PBC-associated renal impairment. By analyzing different types of renal abnormalities reported in patients with PBC, we emphasize that renal involvement should be viewed as an uncommon but clinically relevant extrahepatic manifestation, particularly in selected patients with urinary abnormalities, impaired renal function, autoimmune comorbidities, or advanced liver disease. Within the theoretical framework of the “gut-liver-kidney axis,” gut microbiota dysbiosis may influence the kidneys through immune pathways, contributing to the development of PBC-related renal lesions. By integrating the “gut-kidney axis” and “gut-liver-kidney axis” mechanistic models with PBC’s characteristic bile metabolism abnormalities and hepatic immune features, we propose the following hypothesis: Gut microbiota dysbiosis and bile/metabolic disturbances in PBC patients may induce or exacerbate renal injuries—including glomerular and tubulointerstitial damage—through systemic metabolic, immune, and toxic pathways. Evidence indicates that immune complex-mediated glomerular diseases, renal tubular acidosis, and Fanconi syndrome can all occur in PBC patients. Therefore, early screening for renal impairment and the development of individualized treatment plans are essential in clinical management. Although the diagnostic value of renal biopsy is well established, procedure-related risks represent an important clinical consideration that must be evaluated.

Although current research has revealed the association between PBC and renal impairment, several limitations persist. First, the overall number of cases with PBC and concomitant renal impairment remains relatively small, with studies largely relying on retrospective analyses. Additionally, the precise mechanisms underlying different types of renal lesions have not been fully elucidated. In particular, comprehensive and systematic epidemiological data are lacking, preventing accurate assessment of the true incidence, prevalence, and demographic distribution. Second, while existing treatments are effective for some patients, their clinical application remains controversial. Specifically, the indications and duration of corticosteroid and immunosuppressive therapy remain unclear, necessitating further exploration of personalized treatment strategies.

Future research should focus on the following areas: First, conducting multicenter, prospective cohort studies with larger sample sizes to better understand the pathophysiological mechanisms of PBC-associated renal impairment, with particular attention to the interactions between gut microbiota and immune dysregulation. Second, exploring specific biomarkers for PBC-related renal injury to advance early diagnosis and personalized treatment. Third, actively developing novel targeted therapies, especially evaluating the potential of biologics for PBC-related renal impairment, to achieve more precise and effective treatment. These efforts hold promise for improving the prognosis and quality of life for patients with PBC and renal impairment.

In conclusion, research on PBC-related renal impairment continues to evolve. Despite numerous challenges, emerging research findings and therapeutic approaches will provide richer tools and strategies for clinical diagnosis and treatment, further advancing progress in this field.
